# Neuroendocrine pathogenesis of perimenopausal depression

**DOI:** 10.3389/fpsyt.2023.1162501

**Published:** 2023-03-30

**Authors:** Yuping Han, Simeng Gu, Yumeng Li, Xin Qian, Fushun Wang, Jason H. Huang

**Affiliations:** ^1^Department of Psychology, Medical School, Jiangsu University, Zhenjiang, China; ^2^Institute of Brain and Psychological Sciences, Sichuan Normal University, Chengdu, Sichuan, China; ^3^Department of Neurosurgery, Baylor Scott and White Health, Temple, TX, United States; ^4^Department of Surgery, Texas A&M University, Temple, TX, United States

**Keywords:** perimenopausal depression, three primary color, emotion, neuroendocrine, life stress, therapy, MDD, PMD

## Abstract

With the development of social economics and the increase of working pressure, more and more women are suffering from long-term serious stress and showing symptoms of perimenopausal depression (PMD). The incidence rate of PMD is increasing, and the physical and mental health are seriously affected. However, due to the lack of accurate knowledge of pathophysiology, its diagnosis and treatment cannot be accurately executed. By consulting the relevant literature in recent years, this paper elaborates the neuroendocrine mechanism of perimenopausal depression from the aspects of epigenetic changes, monoamine neurotransmitter and receptor hypothesis, glial cell-induced neuroinflammation, estrogen receptor, interaction between HPA axis and HPG axis, and micro-organism-brain gut axis. The purpose is to probe into new ways of treatment of PMD by providing new knowledge about the neuroendocrine mechanism and treatment of PMD.

## Introduction

Major depression disorder (MDD) is one of the leading causes of disability and premature death around the world. The incidence rates of chronic physical injury and death due to MDD are increasingly high (1). It is estimated that more than 300 million people worldwide suffer from MDD, equivalent to 4.4% of the world’s population (2). It brings great pain to patients and their families, and great economical pressure on the society. Women are twice more likely to suffer from depression than men (3), and it is estimated that approximately 20% of women experience severe depression in her life (4). It can occur or worsen during certain physiological periods with large hormone changes, such as premenopausal, perinatal, perimenopausal and postmenopausal (5). Compared with premenopausal and late postmenopausal women, the incidence of severe depression in perimenopausal women increased by two to three times (6).

The reason why perimenopausal women are prone to depression is unclear, which may be related to various neurological, endocrine, genetic, behavioral and social factors (7). Many studies have proposed different mechanisms and hypotheses, such as the estrogen withdrawal hypothesis, which suggested that estrogen deficiency directly leads to depression (8). However, many other factors are also involved, for example, Han et al. (9) pointed out that it may be related to lower education level, more severe perimenopausal symptoms and cognitive change. Gordon (10) proposed that the fluctuation of tetrahydroprogesterone was related to Regulation of γ-aminobutyric acid. De Kruif (11) held that vasomotor symptoms led to chronic sleep interruption, which leads to irritability and depression. This article will focus on the life stress and its effects on neuroendocrine changes, the relevant evidence and neuroendocrine mechanism of the increased risk of PMD.

### Symptoms of perimenopausal depression

**Figure 1:**
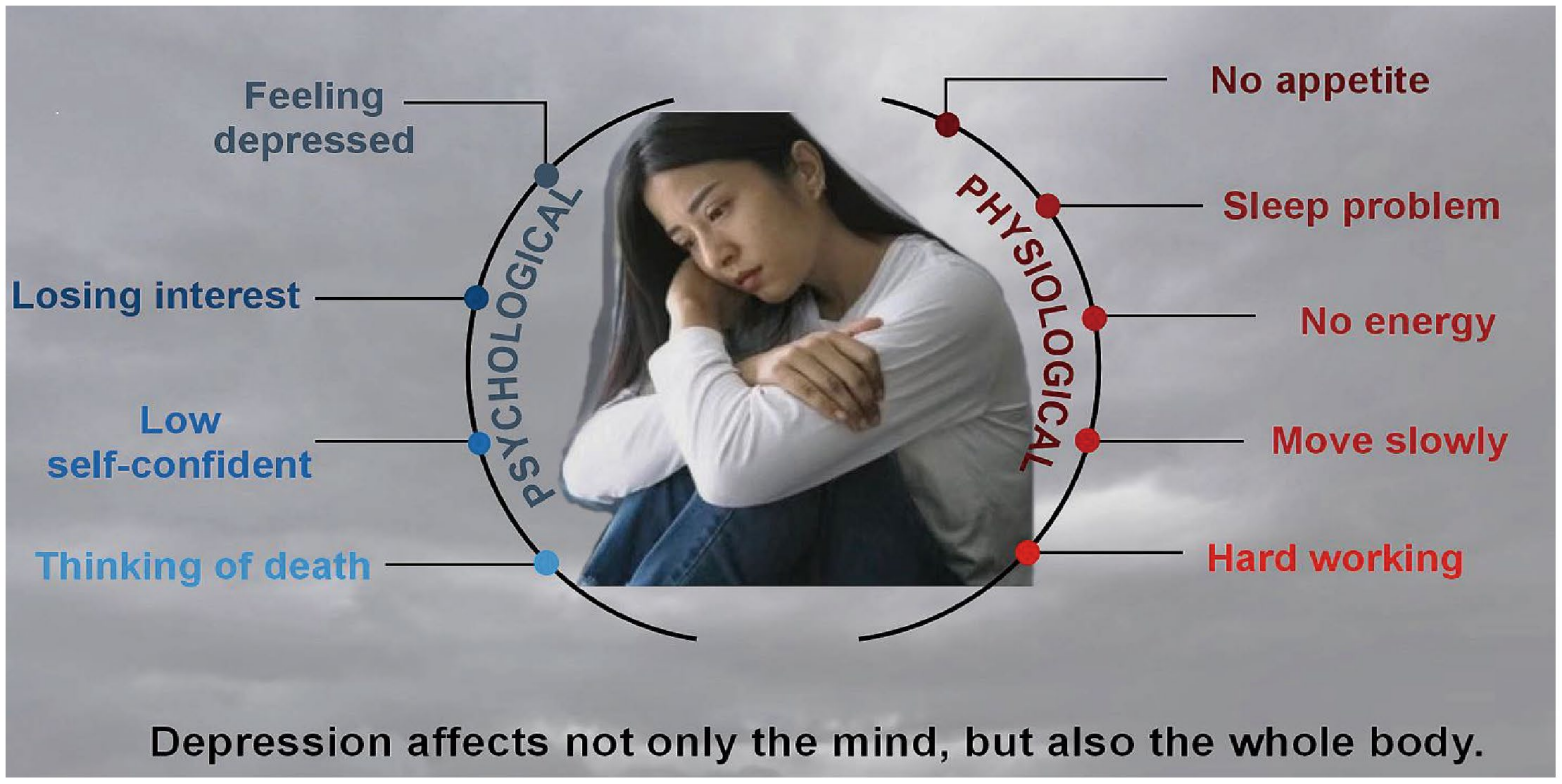
Signs and symptoms of PMD.

The period of perimenopause represents the transition from reproductive life to non-reproductive life, which is a critical physiological transition period for women, with ovarian recession and endocrine disorder. However, not every menopausal woman suffers from PMD, only about 23.8% menopausal women are affected by depression, which suggested that there are several more reasons for PMD in addition to estrogen withdraw. According to the criteria of the seminar on reproductive aging stage, perimenopause is defined as the time span between the first major change in the length of menstrual cycle (the change with a difference of more than 7 days from the individual’s normal cycle length) and 12 consecutive months of amenorrhea (12). More than 80% of perimenopausal women show various physiological and psychological symptoms due to changes in sex hormones (13), such as depression and no interest, sleep disorders, tired, low energy, no self-confidant and thinking of death (Figure 1) (14). Women with anxiety sensitivity are more likely to suffer from vasoconstriction symptoms (15), and some will also suffer from mental disorders (16). Multiple menopausal symptoms occur at the same time and overlap with emotional disorders (17), leading to an increased risk of new or recurrent depression (18).

### Life stress and perimenopausal depression

Women in the menopausal transition and early postmenopausal period have a higher risk of depressive symptoms and negative emotions than those in the postmenopausal period, which may be due to a series of bio-psycho-social risk factors (19). Stress has been suggested to be the major reason for depression. Early life stress can affect one’s whole life, and induce depression in later lives, such as menopause (20). In addition, a large number of clinical studies and animal experiments have shown that chronic stress can lead to neuroendocrine disorders. When stress stimulation persists, glucocorticoids are continuously released and at a high level, which will lead to abnormal release of monoamine neurotransmitters in the brain (21). For example, previous study in our lab has shown that stress can induce changes of hydroxylamine, L-glutamine, L-tyrosine and 3-phosphoglycerate occurred in the hippocampus and intestine of rats with unpredictable chronic mild stress-induced depression (22). Other hormones are also related to depression, for example, melatonin which is release by pineal body, is a strong positive correlation factor between climacteric symptoms and depression intensity.

Recently, genetic studies found that early life stress can induce DNA expression changes which might affect adult behavioral phenotypes (23). Epigenetic changes can make the same genome to expression differently, due to DNA methylation, histone modification, and miRNA etc. It is really the case the early life traumatic events can induce epigenetic changes for many MDD related neuromodulator receptors or transporters, such as methylation DNA of MAO (monoamine oxidase) or HPA axis, or through miRNA and lnRNA (24). The study of MAOA epigenetics found that depression may occur when MAOA is subjected to disordered DNA methylation programming, and MAOA-genotypic variants may mediate NR3C1’s metabolism (25). Recent studies also suggested that epigenetic changes in estrogen receptors are also related to MDD. E2 causes extensive epigenetic changes, mainly through DNA methylation to change gene transcription, such as methylation of genes (such as Est1, Cacna1c and Dcc) related to stress sensitivity and mental disorders (including human MDD) with significant differences (26). In addition, E2 changes the mRNA and protein expression of DNA methyltransferases (DNMTs) (27). In general, estrogen-receptors (ERs) combine with estrogen-responsive elements (EREs) in the nucleus and induce gene transcription. ERα plays a role in passive demethylation through ERE-mediated inhibition of DNMT1 expression. Although the ligand ER binds to the ERE in the DNMT1 promoter, it does not start gene transcription, but inhibits other transcription factors to start the gene expression of DNMT1 (28). Long-term stress stimulation will lead to epigenetic changes in monoamine oxidase receptor or estradiol receptor, increasing the risk of depression. Similarly, due to the biphasic stimulation of physiological changes and social pressure, the genome expression of perimenopausal women is abnormal, and the risk of PMD is increased.

## Prevalent mechanisms of perimenopausal depression

### Monoamine neurotransmitter and receptor hypothesis

**Figure 2:**
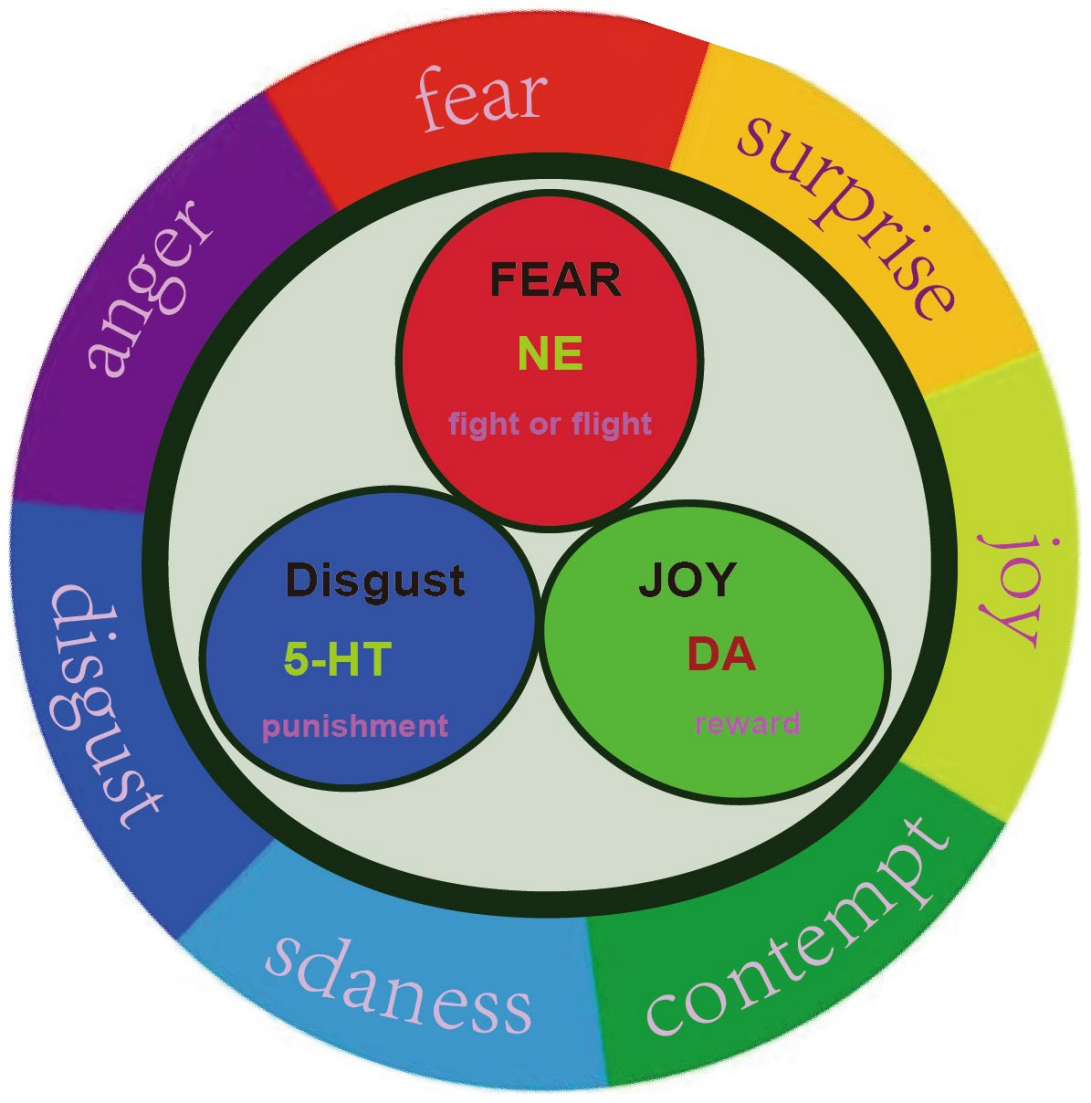
Three primary basic emotions. Russell proposed that all emotions can be located on a circle, instead of a quadrant. We proposed that there might be three primary emotions (Joy, fear, and sadness), adopted from our previous paper (30).

PMD is a unique subtype of depression during perimenopause, and some of its neuroendocrine characteristics are consistent with depression. The hypothesis of monoamine neurotransmitters and receptors is a very important hypothesis for the pathogenesis of depression (24). At present, the first-line treatment for MDD are still antidepressants that have evolved from the monoamine hypothesis. Monoamine neurotransmitters are mainly secreted by the brain and adrenal gland, such as dopamine (DA), norepinephrine (NE) and 5-hydroxytryptamine (5-HT), and play an important role in brain development, emotion regulation, stress, etc. In our previous studies, we originally proposed that there might be only three primary emotions, which are subsided by these three monoamine neurotransmitters (23). The neurotransmitter hypothesis believes that the decrease of monoamine neurotransmitter level in the brain will lead to depression (29). In our hypothesis, we originally differentiate the functions of these three monoamine, and we were the first to propose that norepinephrine is related to stress, dopamine is related to joy, while serotonin is related to depression (Figure 2). Thus we hypothesized that the three monoamine work differently to make three distinct emotions, as in the three primary colors (31). Therefore, monoamine might be the primary substrate for emotions; thus we introduced a new emotional theory based on the three monoamines, which can be called “three primary color model of emotions” (31). The traditional hypothesis proposed that the decrease of 5-HT in the limbic system and cerebral cortex of stroke patients may be an important factor of MDD (32). For example, Pestana-Oliveira et al. (33) found that the level of 5-HT in the amygdala of PMD rats treated with 4-Vinylcyclohexen Diepoxide was lower than that of the control group. However, we proposed that the release of 5-HT might be related to sleep and calm or depression (23).

Alternatively, monoamine decrease in the stress induction experiment of PMD rats, might be related with the release of luteinizing hormone after ovariectomy. In addition, it is observed that 5-HT deficiency in the basolateral amygdala of PMD mice increased glutamate release and inhibited γ- the release of aminobutyric acid leads to anxiety-like behavior. According to the hypothesis of monoamine neurotransmitter hypothesis, different findings have been made in the treatment of perimenopausal patients. He Z et al. (34). believed that the decrease of dopamine D3 receptor alone in the nucleus accumbens accompanying perimenopause was not enough to induce depressive behavior, while the decrease of D3 receptor was greater under the combined effect of stress, which could induce depressive behavior during perimenopause. Xiao M et al. (35) pointed out in a randomized controlled experiment that Huolisu Oral Solution can reduce the depressive behavior of depressed rats and increase the levels of DA, 5-HT and NE in hippocampus and serum. Bhatt (36) have shown that 5-HT_3_ receptor antagonists are effective in treating the comorbidity of depression and anxiety, and can reduce the symptoms of depression and anxiety in rodent models. Amin (37) used fluoxetine and 7,8-dihydroxyflavone to optimize the integration scheme, which can effectively reverse the depressive behavior of perimenopausal mice. In all, monoamine neuromodulator seems to be out dated because the traditional antidepressants are not effective in about one third of MDD patients (38).

### Neuroplasticity hypothesis

The neuroplasticity hypothesis points out that the changes of neuroplasticity caused by stress and other negative stimuli play an important role in the occurrence and development of depression (39), and lead to the occurrence of depression by changing the hippocampal structure, increasing or decreasing neuronal apoptosis or regeneration, signal pathway disorder and synaptic plasticity damage (40). In a group of PMD animal experiments, it was found that the hippocampal pyramidal cell layer of rats in the model group became thinner, the gap increased, the structure was incomplete, a large number of cells were missing, and the ultrastructure of the hippocampus had obvious pathological changes, indicating that the spatial learning and memory of rats in the model group decreased, which was related to the pathological changes in the hippocampal structure (41). Ge (42) induced activation of microglia and inflammatory reaction in the prefrontal cortex of ovariectomized rats can accelerate anxiety and depression mediated by chronic stress. Yao (43) regulated the IL-4R/JAK1/STAT6 signal pathway through astragalus, reduced the activation of microglia in the dentate gyrus, increased ki67 positive cells, and alleviated depression behavior and memory deficits. Zhao et al. (30) found that scutellaria baicalensis can up-regulate TGFβ. The level of p-SMAD2/3 and NEDD9 protein and the increase of the number of DCX-, MAP2- and NeuN positive cells in the hippocampus indicate that scutellaria baicalensis can mediate TGFβ3-Smad2/3-Nedd9 signal pathway protects neurons to improve depressive behavior. Jing et al. (44) treated PMD model rats with electroacupuncture therapy of “combination of kidney and brain” for 28 days. Compared with clomipramine hydrochloride drug group, in the experimental group, the content of estradiol and dopamine increased, the expression of DKK-1 increased, the expression of LRP-5 and LRP-6 decreased in the hippocampus, and Wnt β- Catenin signal pathway is inhibited, thus promoting the repair of nerve cells and improving the symptoms of depression, but acupuncture has less side effects than drugs.

### Changes of brain-derived neurotrophic factor level

The level of brain-derived neurotrophic factor (BDNF) in peripheral serum is closely related to the severity of depression (45). When patients have depressive symptoms, the content, synthesis and release of BDNF in peripheral blood serum are significantly reduced (46). This change is of great significance for the diagnosis and treatment of perimenopausal syndrome (47). In a clinical controlled trial, Xue (48) found that the BDNF level of perimenopausal depression patients who took venlafaxine capsule increased, the HAMD score and depression symptoms improved significantly, and the good or bad drug effect was related to the BDNF level. Chaihu has been used in clinical treatment of PMD for a long time. It mainly regulates the expression of neurotransmitters and the abnormality of ERK1/2-CREB-BDNF signal pathway (49), so as to improve the behavioral changes and inhibit the depression of perimenopausal rats with liver depression. Both Chaihu Shugan San (50) and Saikosaponin (51) can promote the activation of BDNF – TrkB signal pathway in the hippocampus and mediate the recovery of the neurotrophic system to produce antidepressant-like effects.

### Neuroglia cell and glial lymphatic system

The brain is the only organ without lymphatic system, but the blood–brain barrier (BBB), special immune cells and the system connecting the brain and peripheral circulation are known as glymphatic system (52), which enables the brain to respond to injury as sensitively and accurately as the immune system. Many nervous system diseases, such as severe depression, autism, Alzheimer, Parkinson’s disease and multiple sclerosis, are manifested as aggravated inflammation or incorrect response of central nervous system immunity (53).

Microglia respond to stress-induced neuroinflammation by releasing pro-inflammatory cytokines (TNF-α, IL-1β, IL-6) and its metabolites (54), meanwhile, the excessive activity of neurons triggers ATP release through activation of N-methyl-d-aspartic acid receptor and microglia ATP receptor to recruit microglia (55), leading to a series of reinforcement reactions, damaging the nerve function and leading to depression. Interferon-gamma(IFN-γ) The activation of microglia damages the neurogenesis of adult hippocampus and leads to depressive behavior and cognitive impairment (56). Microglia regulate inflammation, synaptic plasticity and the formation of neural network, which will affect depression, so some people think that depression can be regarded as a microglial disease (57).

The glymphatic system is dependent on the activity and polarization of aquaporin 4 (AQP4) on the terminal foot of astrocytes to mediate the exchange and flow system of cerebrospinal fluid and brain tissue fluid, and to mediate the excretion of macromolecular substances in brain tissue fluid (58). There are abnormal or decreased astrocytes (59, 60), depolarized AQP-4 (61) and dysfunctional glymphatic system (62) in emotional disorders. The expression of messenger RNA (mRNA) transcripts involved in AQP4 expression in MDD patients is down-regulated (63), and even the pathological conditions of depression may increase the risk of Alzheimer’s disease development through the impairment of glial lymphatic pathway function (62). The decrease of astrocyte density can also be transmitted to the offspring of depressed females through epigenetic mechanism (64). Liu et al. found that the supplementation of polyunsaturated fatty acids improved the physical signs of depression and the accompanying cognitive dysfunction by restoring the potential damage of the glymphatic system and protecting the cerebrovascular function (65). However, the study found that when iron accumulation and chronic stress exist simultaneously, the expression of transferrin receptor in neurons increases, and the increase of neuronal apoptosis caused by the destruction of glymphatic system by iron metabolism disorder aggravates the depression of stress mice (66). In astrocyte proliferation, astrocytes do not act alone, but together with microglia and neural/glial antigen 2 cells (67). Microglia can activate astrocytes, leading them to enter the pro-inflammatory state (68). To sum up, chronic stress leads to the release of inflammatory factors from microglia and damages the function of glymphatic system induced by neurons and astrocytes, which is the possible cause of depression.

### Endocrine mechanism of perimenopausal depression

The above discussion on the neurosecretory mechanism of PMD is based on depression, mainly including the hypothesis of monoamine neurotransmitters and receptors, the hypothesis of neural plasticity, and the hypothesis of neurotrophic molecules. However, they cannot explain the phenomenon that depression much more easily occurs in female, and the gender difference in MDD are attracting more and more attention from the scientists (69). Many studies indicate that, although girls are no more depressed than boys in childhood, more girls than boys are depressed by ages 13 to 15. The onset time of this sex bias has been attributed to a wide variety of factors, such as emotional regulation, rumination, cognitive style or temperament, or pubertal hormones. Even though the list of possible causal factors has been reviewed previously, actually they are possibly derived from changes of sexual hormones. For example, perimenopause is a more complex process of internal environment, mainly manifested in the decline of ovarian function, decreased estrogen secretion, and increased secretion of luteinizing hormone (LH) and follicular estrogen (FSH) (70). Estrogen can regulate the transcription and expression of target genes after binding with receptors, which can affect the synthesis of monoamine neurotransmitters, promote the growth of neurons, inhibit their apoptosis, and regulate the signal pathway of brain-derived neurotrophic factors (71, 72). It shows that PMD is the result of the changes of neuroendocrine.

### Changes of hypothalamus-pituitary-gonad axis

The HPG axis is the neuroendocrine axis that drives and guides the reproductive function. The hypothalamus secretes gonadotropin-releasing hormone(GnRH) to act on the pituitary, and the pituitary secretes FSH and LH to stimulate the production of estrogen and progesterone and drive the growth and maturation of germ cells. The reproductive function of women is cyclical.

Estrogen is a major female hormone, including estrone and estradiol, which are mainly produced by the ovary and placenta. The great role of estrogen in women’s life cannot be replaced by any hormone. It dominates the development and maintenance of women’s secondary sexual characteristics, regulates the stability of women’s internal environment, controls women’s life cycle, and women’s periodic menstruation, women’s fertility, and women’s unique body shape cannot be separated from the role of estrogen. In addition to the reproductive system and estrogen, many tissues and organs in women have their target organs, such as the nervous system, cardiovascular system, bone, urinary system, and so on. LH is a gonadotropin secreted by the pituitary gland, which promotes the conversion of cholesterol in gonadal cells into sex hormones. For women, FSH works together to promote the maturation of follicles, secretion of estrogen, ovulation, formation and maintenance of corpus luteum, secretion of progesterone and estrogen. FSH is a hormone secreted by basophils in the anterior pituitary gland. Its component is glycoprotein and its main function is to promote follicular maturation. Follicle-stimulating hormone can promote the proliferation and differentiation of granulosa layer cells and promote the growth of ovary.

For healthy women, the ovaries undergo periodic ovulation and the production and secretion of estradiol/progesterone under the action of FSH and LH. The estradiol/progesterone secreted by the ovaries has a feedback regulation on the synthesis, secretion and release of hypothalamic hormones, and GnRH can also directly inhibit the function of the ovaries (73). This bottom-up and top-down interaction of HPG axis makes female endocrine maintain a relatively stable dynamic balance under normal conditions.

The incidence of depression in women is more than twice that in men (3), indicating that estrogen is related to the incidence of depression (74). The secretion of estrogen decreases during perimenopause, and the incidence rate of depression is two to three times that before menopause (6), indicating that estrogen deficiency will lead to depression (12). The two views are diametrically opposite. During perimenopause, changes in physiology, emotion, psychology and society mark the development of women from childbearing to menopause. No matter from the number of follicles or the quality of oocytes, the ovaries gradually fail, the sex hormone fluctuates greatly, and the negative feedback effect of the hypothalamus-pituitary-gonad axis also weakens (75), resulting in an increase in the secretion of LH and FSH (70), until the onset of hypergonadotropic hypofunctional amenorrhea. Whether women are in the early or late menopause, the highly variable and unpredictable reproductive hormone dynamics during perimenopause at least partially explain the variability of depressive symptoms (76). Next, we will discuss the role of estrogen, progesterone and other sex hormones in the pathogenesis and treatment of PMD.

#### Estradiol

Estradiol has a profound impact on the chemistry, structure and function of the brain, and has a nutritional effect on the prefrontal cortex and hippocampus, which are important for regulation and cognition (77). Estrogen stimulates serotonin activity by increasing the number of serotonin receptors and the transport and uptake of neurotransmitters (78). A considerable number of women show moderate and high emotional sensitivity to changes in endogenous estrogen during the menopausal transition period, and the emotional sensitivity to estradiol indicates the level of PMD risk (79). Excessive menstruation and fluctuating estradiol levels affect the binding of monoamine oxidase A (80), and increase the risk of severe depression in women by affecting the stress response and emotional regulation in the brain network. Therefore, estradiol treatment needs to be considered in the early menopausal transition period before the decline of ovarian hormone level permanently affects the serotonin function (77). Kulkarni (81) found in a 12-week randomized controlled trial that oral tibolone could improve the depression score of perimenopausal women without any significant side effects. Another study proved that the experimental group (receiving 12 weeks 17 β- Estradiol transdermal patch) is superior to placebo in the treatment of PMD (82). The effect of estrogen on the psychological status of patients with depression and schizophrenia fluctuates, but the overall trend is improving (81). However, there are many clinical studies show that hormone replacement therapy has little effect on PMD (83). For postmenopausal women without a history of severe depression, taking estrogen leads to decreased activity in the inferior frontal lobe, decreased emotional regulation function, and more negative emotional reactions to psychosocial stress (84). Demetrio (85) and Girdler (86) found that the BID score of oral estrogen replacement therapy did not improve in postmenopausal women. In a randomized controlled experiment, subjects were randomized to receive percutaneous estradiol, oral zolpidem or placebo treatment for 8 weeks after a one-week running-in period. The score of the Montgomery Depression Scale was improved in all groups, and the sleep quality of menopausal and postmenopausal women was improved (87). Depression is a psychological disease, and patients’ psychological cues to themselves are also extremely important, so it cannot be concluded that the improvement of depression score is the effect of hormone therapy. To sum up, the efficacy of estrogen replacement therapy on PMD is controversial, and the side effects of estrogen on breast cancer, endometrial cancer, cardiovascular disease (88) cannot be ignored. Therefore, whether estrogen replacement therapy can be used as a clinical first-line treatment remains to be discussed.

However, recent studies have found that E2 has little effect on perimenopausal depression. Female depression induced by estrogen deficiency may be related to 5-hydroxytryptamine (5-HT) deficiency. In addition, luteinizing hormone (LH) changes significantly in menstrual cycle, perinatal period and perimenopause, which may be the main cause of depression (89). Before ovulation, LH secretion can reach 3–8 times of the basic level, and during perimenopause, LH secretion can increase to 3 times of the previous level (90). LH and FSH are positively correlated with the changes of stress hormones such as cortisone and adrenocorticotropin. LH and FSH may be specific risk factors for the development of PMD (91).

#### Estrogen receptors

**Figure 3:**
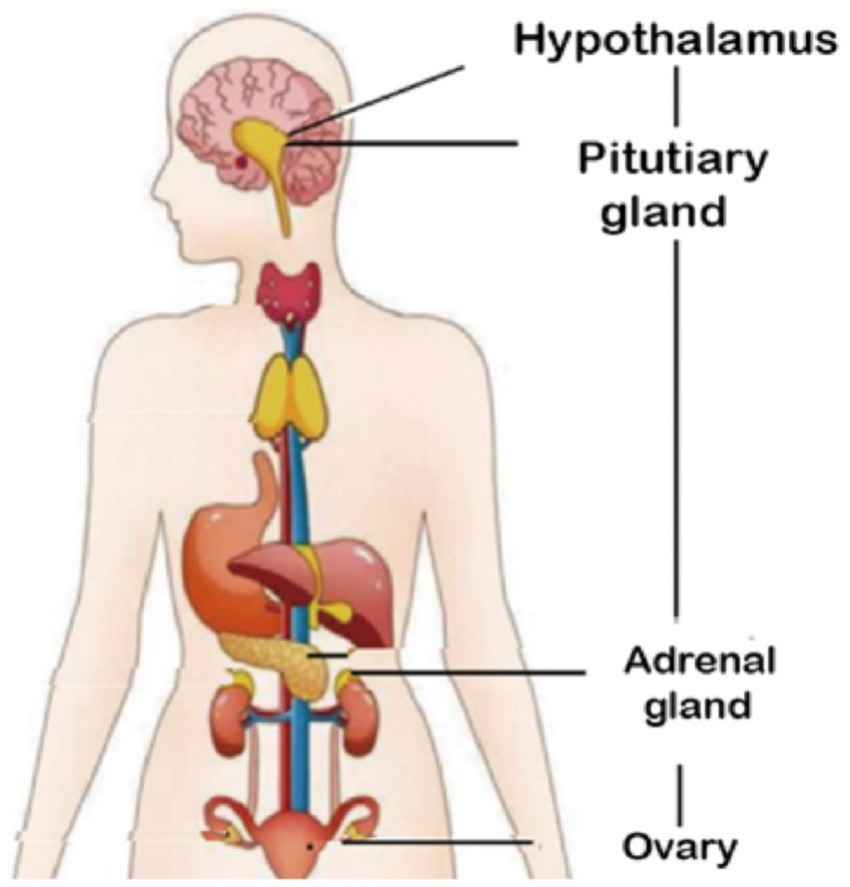
HPG and HPA axis.

Estrogen receptors are distributed in different brain regions (such as the medial amygdala, hippocampus and limbic system), and many studies have suggested that they can affect many types of glia cells (Figure 3) (92). There are three known receptors, estrogen receptor α (ERα) Estrogen receptor β (ERβ) and g-protein-coupled estrogen receiver (GPER). ERα and ERβ are composed of different functional domains, and have several common structural regions, including N-terminal terminal domain (NTD) and estrogen response element (ERE). Estrogen can play a direct role by entering the plasma membrane, bringing the estrogen receptor complex to the nucleus, and interacting and binding with the ERE of ERα and ERβ in the cell. In addition, estrogen can also play an indirect role by interacting with receptors to activate various intracellular signal pathways such as PI3K/Akt, ERK or Jak/STAT (93). Therefore, estrogen signals can be divided into genome (directly binding to ERE) and non-genome (activating intracellular signal cascade).

Estrogen receptors widely exist in neurons and glial cells, and bind to receptors through estradiol and play a neuroprotective role (94). The anti-inflammatory effect of estradiol on astrocytes is mainly mediated by regulating the nuclear factor NF-γB of activated B cells (95). In the Lipopolysaccharide (LPS) – induced depression model, it was found that E2 or ERα agonist treatment inhibited the activation of NF-γB and reduced the expression of pro-inflammatory cytokines, indicating that NF-γB and downstream inflammatory cytokines were regulated by E2/ERα (96). Estradiol can also inhibit the transcription of NF-γB dependent cytokines such as CCL2 by activating ERα in astrocytes (97). It can also play an anti-inflammatory role by increasing the release of growth factors such as IGF-1 and reducing the release of Ca^2+^ (98). Some studies have found that two gene variants of ERα gene (ESR1) (rs22346939 and rs9340799) are associated with MDD risk and its characteristics in individuals and combinations (99). Like astrocytes, estradiol binds to ERα of microglia and inhibits transcription factor NF-γB through activation of PI3K (100). On the other hand, estradiol down-regulates the gene expression of plasma membrane monoamine transporters (PMAT, Slc29a4) through ERβ and MAPK/ERK signaling pathways, thereby reducing the reuptake of 5-HT (101). Estradiol can also increase GABA levels in hippocampus and frontal cortex through ERβ and/or GPR30, and up-regulate GABA-related genes in amygdala and hippocampus (102). Estradiol also binds to ERγ and GPER1/GPR30 microglial receptors, thereby regulating the release of inflammatory mediators and reducing the activation of microglial cells (103). The above researches show that ERβ activation has antidepressant effect, and the research report on the increase of anxiety-like behavior in ERβ gene knockout mice (104) further supports this view. GPR30 or GPER1 is a new estrogen receptor found in recent studies (105, 106). GPR30 is widely distributed in the brain of rodents, and high levels of immunoreactivity are found in areas related to emotional behavior, such as cortex, hippocampus, hypothalamus and brain stem (107). The activation of GPR30 can lead to the activation of adenylate cyclase, stimulate the production of cAMP, and finally activate the transcription factor cAMP-response element binding protein (CREB) (108). Estradiol can activate extracellularly signal-regulated kinase (ERK) through GPER1, and then mediate the increase of synaptic transmission (109). Tian et al. (110). proposed that GPR30 regulates anxiety-like behavior by changing the balance between GABAergic and glutamatergic signals in the basolateral amygdala, and the activation of GPR30 increases the inhibitory synaptic transmission in the basolateral amygdala of ovariectomized mice. The above discussion mainly focuses on the contribution of each estrogen receptor in anxiety and anxiety-like behavior, providing theoretical support for hormone replacement therapy of perimenopausal depression.

#### Progesterone

In addition to the wide fluctuation of estradiol during perimenopause, the changes of gonadal hormones also include the reduction of the frequency of progesterone production during anovulation. The potential independent effect of progesterone on mood may be mediated by its neurosteroid metabolite tetrahydroprogesterone (10). As a neurosteroid, it may directly inhibit γ- Aminobutyric acid receptor, which mediates the protective effect of peripheral progesterone on mood, thus has a beneficial effect on the mood of women with hormone-related mood disorders (69). Tetrahydroprogesterone has been approved by the US Food and Drug Administration for the treatment of postpartum depression (111). The use of estrogen alone increases the risk of endometrial thickening and endometrial tumor (112). Progesterone can cause endometrial abscission, interfere with the binding of estrogen and receptor, and thus offset the proliferative effect of estrogen on uterine tissue (113). Progesterone is used in combination with hormone replacement therapy to stabilize endometrial tissue and reduce estrogen side effects. Sovijit (114) found in ovariectomized depressed mice that progesterone can reduce depression and anxiety by regulating the changes of intestinal microbiota composition, especially increasing *Lactobacillus* population. Hormone therapy is usually used to relieve depression and anxiety symptoms, but the health risks of estrogen, progesterone or synthetic progesterone treatment are greater than their benefits (89). However, progesterone is effective in improving depression symptoms and safer for menopausal women (115).

### Changes of hypothalamus-pituitary-adrenal axis

HPA axis is an important endocrine system, responsible for coordinating stress response, which is a complex set of behavioral, neuroendocrine, autonomic and immune responses, and making appropriate response to stressful life events (116). When stimulated by external pressure, the paraventricular nucleus of the hypothalamus (PVN) synthesizes and secretes adrenocorticotropin-releasing hormone(CRH), stimulates the pituitary to synthesize and release adrenocorticotropic hormone(ACTH) into the blood, and after reaching the adrenal cortex, sends out the signal of glucocorticoid synthesis (117), glucocorticoid is released into the blood to promote the proper response of the body to environmental disturbance. The HPA axis is finally inhibited by the same hormone acting on different regions of the brain, and this negative feedback process reconstructs the baseline steady-state (118).

The abnormality of hypothalamus-pituitary-adrenal (HPA) axis is a common neuroendocrine abnormality of PMD, which is characterized by the persistent stress state of the body for a long time, and the continuous high level of serum glucocorticoids, ACTH and CRH (119). Compared with men, the HPA axis imbalance caused by chronic stress in women is more serious, which is manifested by a significant increase in glucocorticoids and ACTH (120). This difference is mainly due to the regulation of gonadal hormones such as estradiol (121). Gonadoxins can interact with the central and peripheral components of HPA axis to regulate the synthesis and release of CRH, ACTH and glucocorticoids (122). Li (123) found that Orcinol Glucoside (OG) can improve the dysfunction of HPA axis caused by estrogen withdrawal and hormone fluctuation, alleviate the hyperactivity of HPA axis by regulating the levels of corticosterone, ACTH and CRH, and strengthen the BDNF-TrkB-CREB signal pathway in the hippocampus, which has potential antidepressant effect. Erxian decoction has neuroprotective effect on PC12 cells damaged by corticosterone, and can effectively inhibit reserpine-induced hypothermia, ptosis and dyskinesia in mice. It has antidepressant-like effect, which may be related to the regulation of monoamine nerve transposition system in the brain (124). Contrary to the traditional idea, Guerrierieri GM (125) tested the HPA axis function of perimenopausal women with and without depression using the combined dexamethasone-adrenocorticotropin releasing hormone test. It was found that there was no inter-group difference in both baseline and stimulated ACTH and cortisol secretion. No abnormal HPA axis activity related to PMD was observed, indicating that PMD and HPA imbalance were not consistent. It may reflect the different underlying pathophysiological processes of patients with non-productive depression.

#### HPA and HPG

Because the lipophilicity of peripheral steroid hormones can cross the brain blood barrier, they can be transformed into bioactive metabolites in the brain through the action of aromatase, 3β-HSD, and 5α- reductase (126), interact with receptors to regulate cell function. For example, the effect of testosterone on the brain is usually controlled by the conversion of testosterone into estradiol by aromatase (118). 5α- Reductase converts progesterone and glucocorticoid into their respective metabolites in the brain (127). 3β- Diol plays a role by binding and activating ERβ (128). E2 can reduce the response of ACTH to stress (129). One study found that the incidence rate of depression in some women with oral contraceptives increased, which may be due to the fact that the contraceptives inhibit the production of endogenous steroids such as estradiol, which destroys the HPG axis and thus affects the regulation of the HPA axis (130). These data indicate that estradiol is an important inhibitor of female hypothalamus-pituitary–adrenal stress response. However, other experiments have shown that E2 can increase stress-induced neuronal activation (131) and CRH gene expression (132), and increase the sensitivity of adrenal gland to ACTH (133). The report on the obvious adverse effect of E2 on HPA axis activity may be due to different experimental conditions, such as the dose or duration of E2 treatment, on the other hand, it may be E2 on ERα and ERβ (ERβ Decrease, while ERα Increase HPA axis gain) mediated nondifferential binding of signal transduction (134). At the same time, this may explain the phenomenon that estrogen plays different roles in women’s depression at different periods and has different incidence rate.

At the same time, glucocorticoids have inhibitory effects on different levels of HPG axis, such as thalamus (reducing the synthesis and release of GnRH), pituitary (inhibiting the synthesis and release of LH and FSH), testicle/ovary (directly regulating steroid production and/or gametogenesis) (135). The central neuropeptide and CRH of HPA axis significantly inhibit the activity of HPG axis at the central level (136). CRH inhibits the HPG axis at the central and pituitary levels through CRHR1 and CRHR2, respectively (137). Infusion of CRH receptor antagonists can reverse the inhibition of LH level induced by acute stress. HPG axis is easily affected by stress, which has a strong impact on estrus cycle, GnRH/LH activity and fertility (138). To sum up, in order to improve the survival rate and quality of life, HPA and HPG axes work together and fine-tune each other, so as to integrate environmental, psychological, reproductive and genetic factors. Problems in any of the regulatory systems will cause the disorder of body functions.

Cushing’s syndrome (CS) is a rare endocrine disease. The excessive secretion of ACTH caused by pituitary adenoma or HPA axis dysfunction stimulates bilateral adrenal cortex hyperplasia, resulting in excessive production of chronic glucocorticoid (GC). In addition to typical clinical symptoms (full moon face, centripetal obesity, hypertension, etc.), there are also severe depression, mania, anxiety and neurocognitive disorders (139). GC plays a crucial role in stress response. GC receptors have pleiotropic distribution in the central nervous system (CNS), mainly in the hippocampus. GCs induced brain injury put forward four hypothesis mechanisms – decreased glucose intake leads to brain atrophy, increased excitatory amino acid toxicity on nerve cells, inhibition of “long-term enhancement” leads to cognitive impairment, and inhibition of dentate gyrus neurogenesis (140). These mechanisms seem to be able to explain the GC-induced brain damage, mainly the hippocampus damage that leads to neurocognitive impairment. After the disappearance of hypercortisolism, although the overall prevalence of mental disorders and neurocognitive disorders has improved, some patients may still show depression, anxiety, panic disorder and neurocognitive disorders, which play a key role in affecting the health and daily life of patients after long-term remission (141). Clinical study found that the continuous increase of LH can induce ACTH-independent Cushing syndrome (142). Our research team found in the previous study that ovariectomized rats showed a synchronous and continuous increase in LH and cortisol in the stress environment (143), while ACTH and cortisol have been proved to be an important mechanism of increased susceptibility to depression. At the same time, depression will continue to stimulate the HPA axis, resulting in its hyperactivity and pseudo Cushing syndrome, which brings many difficulties to the diagnosis of CS (144).

### Changes of microbiota-gut-brain axis

Although the change of neuroendocrine mechanism is considered to be the main cause of PMD, microbiota-gut-Brain axis is also a very important possible mechanism. The communication network between the gut and the central nervous system is complex, including the branches of the enteral nervous system (ENS) (ENS), sympathetic nerve and parasympathetic autonomic nervous system (ANS), and neuroimmune and neuroendocrine signal pathways (145, 146). Microflora plays an active role in the function of the nervous system through the interaction with the gut – brain axis (147). Intestinal microbiota can regulate the synthesis and metabolism of neurotransmitters to affect brain functions, such as the neurotransmission of 5-HT, noradrenergic, dopaminergic, glutamatergic and GABAergic (148), and can also produce these neurotransmitters by itself. For example, Candida, *Escherichia coli*, Enterococcus and Streptococcus belong to serotonin producers (149), Bifidobacterium and *Lactobacillus* produce GABA (150), *Lactobacillus* produces acetylcholine, *Bacillus* and *Serratia* produce dopamine, and *Escherichia coli* and yeast produce norepinephrine (151). The intestinal microbiota of patients with depression is significantly different, and the diversity and richness of microbiota have decreased. At the phylum level, the abundance of Bacteroides and Proteobacteria increased, while the abundance of Thickwallida decreased (152); At the family level, the relative abundance of Prevalenidae increased; At the genus level, the abundance of fecal bacteria and rumen cocci (153), lactic acid bacteria and bifidobacteria decreased (154). First, some experiments have proved that intestinal microbial changes can induce depression. The levels of interferon c (IFN-c) and tumor necrosis factor alpha(TNF-a) in the hippocampus of mice treated with CUMS microbiota were significantly increased and depressive behaviors were observed (155). The protein expression level of several tissues (especially the prefrontal cortex and liver) in the gut microbiota of sterile mice receiving MDD patients changed. These protein changes participated in a variety of biological functions, including metabolic processes and inflammatory reactions (156). Secondly, the symptoms of mood disorder can be alleviated through the transplantation of fecal microbiota (157), which opens up a new idea for the treatment of perimenopausal depression. Nishino et al. (158) showed that the intestinal colonization of symbiotic bacteria in CUMS sterile mice led to the increase of monoaminergic neurotransmission in the striatum, leading to the normalization of anxiety-like behavior. Deng et al. (159) found that Paraacteroides improved the level of adverse metabolites in the Kynsignaling pathway, such as Kyn and 3-HK, and revealed that by affecting the Kyn pathway in the gut brain interaction, it is beneficial to improve the depression and anxiety-like behavior induced by chronic inhibitory stress (CRS), providing potential evidence for the link between intestinal ecological disorders, Kyn signaling pathway and depressive behavior changes. A study have found that ERα protects the host from harmful inflammation and dysfunction of mitochondria through autophagy activation and intestinal microflora control, thus promoting intestinal homeostasis (160). For a long time, antidepressant therapy usually aims at brain abnormalities, while other organ dysfunction is ignored. Regulating intestinal microbiota and improving the function of microbiota-entero-brain axis may have a profound impact on the treatment and prevention of depression (161).

## Conclusion

With the development of social economics, the life stress is increasing, and the incidence rates of mental disorders and depression are also increasing, especially for the menopausal women. The incidence of perimenopausal depression has increased to a very high rate that cannot be ignored. Although various mechanisms are suggested for the PMD process, it seems that ovarian dysfunction might be the key reason that links the factors for PMD (162). Recently, many studies have evaluated the importance of ovarian dysfunction in the risk of PMD as well as some other complications such as cardiovascular disease (163). The pathogenesis of PMD is still unclear. It may be related to various neurosecretions, hormone fluctuations, genetics, psychology and society. This article focuses on two aspects, including changes in neurosecretions and endocrinology. Firstly, long-term stress of perimenopausal women will lead to epigenetic changes of estrogen receptors, neuroinflammation induced by microglia and glial lymphatic system disorder mediated by astrocytes, and increase the risk of PMD. Secondly, estrogen can interfere with monoamine neurotransmitters and GABA metabolism, and the activation of estrogen receptor has a regulatory effect on nerve cells. These effects become unstable with the changes of sex hormones during perimenopause, which may be one of the reasons for the increased incidence rate of PDM. Finally, intestinal microorganisms are the focus of research in recent years. Microorganisms can regulate neuroendocrine through the gut – brain axis. Fecal bacteria transplantation may open a new idea for the treatment of PMD.

Although great progress has been made in the pathogenesis and treatment of PMD, there are still many problems to be solved. First of all, in terms of pathogenesis, the effect of estrogen on female depression (weakened or enhanced) is controversial (12, 74), and the enhancement of HPA axis secretion of PMD has not been observed in some clinical experiments (164). Secondly, in the treatment of PMD, the side effects of estrogen replacement therapy are large (114), and the results are doubtful (84–87). However, depressed menopausal women can benefit from antidepressants. Some people respond well to hormones, and some people need both (17). In addition, estrogen and progesterone need to work together (113) to reduce the side effects of estrogen. Of course, while paying attention to medication, mood stabilizers and psychotherapy will continue to be good methods for treating emotional disorders (165).

## Author contributions

YH, SG, YL, XQ, FW, and JH involved in the writing of the original draft. All authors contributed to the article and approved the submitted version.

## Funding

The article was supported by a grant from National Nature Science Foundation in China (82101602).

## Conflict of interest

The authors declare that the research was conducted in the absence of any commercial or financial relationships that could be construed as a potential conflict of interest.

## Publisher’s note

All claims expressed in this article are solely those of the authors and do not necessarily represent those of their affiliated organizations, or those of the publisher, the editors and the reviewers. Any product that may be evaluated in this article, or claim that may be made by its manufacturer, is not guaranteed or endorsed by the publisher.
